# Perspectives on Team-Based Care in Family Medicine: A Mixed-Methods Study on Interprofessional Collaboration in a Hospital Setting

**DOI:** 10.7759/cureus.81388

**Published:** 2025-03-28

**Authors:** Junya Ohara, Ryuichi Ohta, Chiaki Sano

**Affiliations:** 1 Family Medicine, Unnan City Hospital, Unnan, JPN; 2 Community Care, Unnan City Hospital, Unnan, JPN; 3 Community Medicine Management, Shimane University Faculty of Medicine, Izumo, JPN

**Keywords:** family medicine, general medicine, healthcare quality improvement, interprofessional relations, patient-centered care, rural, team-based care, workforce collaboration

## Abstract

Introduction

Interprofessional team-based care has been increasingly recognized as a crucial strategy for enhancing healthcare quality and efficiency. Team-based care fosters comprehensive, patient-centered care by integrating various healthcare professionals, such as physicians, nurses, pharmacists, and rehabilitation specialists, improving coordination and communication while reducing medical errors. Despite the benefits, challenges such as team composition, workflow integration, and professional role clarity remain, necessitating further investigation. This study examines different healthcare professionals' perspectives on introducing team-based care in family medicine within a hospital setting.

Methods

This mixed-method study was conducted at Unnan City Hospital, a regional hospital in Japan. A total of 220 healthcare professionals, including nurses, therapists, medical technicians, pharmacists, and social workers, participated in a structured questionnaire. The questionnaire assessed perceptions of team-based care regarding consultation ease, coordination, understanding of physicians’ perspectives, patient satisfaction, work efficiency, and stress levels. Quantitative data were analyzed using statistical tests, including chi-square and Kraskal-Wallis analyses, while qualitative responses were thematically analyzed.

Results

Findings indicate that healthcare professionals generally perceived team-based care positively, particularly in enhancing communication and interdisciplinary collaboration. Nurses and therapists reported improved work efficiency and stress reduction, whereas technicians and pharmacists expressed concerns about increased workload and role ambiguity. Social workers demonstrated the most favorable perception, highlighting patient-centered care and teamwork benefits. However, inconsistencies in information sharing, decision-making, and workload distribution were identified as key challenges.

Conclusion

While team-based care enhances collaboration and patient-centered approaches in family medicine, its effectiveness is influenced by professional role clarity and structured implementation. Addressing communication gaps, streamlining decision-making processes, and developing standardized protocols are essential for optimizing team-based care. Future research should assess long-term impacts on patient outcomes and healthcare efficiency.

## Introduction

A team-based approach in hospital settings is crucial for enhancing healthcare quality and efficiency [1,2]. By fostering collaboration among various healthcare professionals, it is possible to combine each expert’s unique knowledge and skills, thereby enabling comprehensive, patient-centered care that improves the safety and quality of treatments [3]. This system not only enhances information sharing and coordination to prevent medical errors and promote efficient workflow, but it also contributes to increased patient satisfaction [4]. Additionally, team-based care provides continuous learning opportunities within the healthcare industry, making it essential for adapting to the ever-evolving demands of modern medicine [4].

The introduction of team-based care in hospital settings has significantly impacted various healthcare professionals [5]. This system provides a platform for integrating the expertise and skills of different professions, such as physicians, nurses, pharmacists, rehabilitation specialists, and technicians [6]. As a result, it strengthens information sharing and communication, providing holistic care beyond individual expertise [7]. Moreover, such interprofessional collaboration promotes each professional’s continuous learning and growth, which is critical in enhancing healthcare quality and safety [8].

The introduction of interprofessional team-based care in family medicine has gained attention to improve the quality and efficiency of healthcare in recent years. Family medicine involves diagnosing and treating various health issues and requires a comprehensive understanding of patients’ biological, psychological, and social aspects [9]. To achieve this holistic approach, it is essential for professionals from various fields, including physicians, nurses, pharmacists, and rehabilitation experts, to collaborate effectively [10-12]. Given this background, there is a growing expectation that implementing interprofessional teams will improve the quality of care in family medicine. Some studies and case reports have indicated that interprofessional teams have effectively communicated and collaborated, improving patient health outcomes and increasing healthcare efficiency [4]. However, the effectiveness and challenges of implementing team-based care can be influenced by factors such as team composition, operational methods, cultural backgrounds, and approaches to education and training. Therefore, further research is needed to comprehensively evaluate the impact of interprofessional team-based care in family medicine and develop practical guidelines and educational programs based on these findings. Through this study, we aim to clarify the perspectives of different healthcare professionals on introducing team-based care in hospital settings within the domain of family medicine.

## Materials and methods

This mixed-method study aimed to clarify the perspectives of different healthcare professionals on introducing team-based care in hospital settings within the domain of family medicine. This study used a convergent mixed-methods design, in which quantitative and qualitative data were collected and analyzed in parallel and then integrated during interpretation. Data from a questionnaire asking about the change in quality of care by implementing team-based care were used.

Setting

In 2022, Unnan City had a total population of 35,738, comprising 17,231 males and 18,507 females. Residents aged 65 years and older made up 40.27% of the population. A single public hospital served the area with 281 beds, categorized into 155 acute care, 48 general, 30 rehabilitation, and 48 chronic care beds [13]. The Department of Family Medicine at this hospital coordinates the care of internal medicine patients in collaboration with various healthcare professionals [14]. This study was conducted at Unnan City Hospital from January 1, 2024, to March 31, 2024.

Participants

The study population comprised various healthcare professionals working at Unnan City Hospital. Specifically, the participants included nurses, rehabilitation specialists, social workers, medical technicians, and pharmacists. These professionals were involved in providing care in the hospital's family medicine department and were selected to represent the multidisciplinary workforce necessary for implementing team-based care. Their perspectives on the introduction and impact of team-based care were gathered through a structured questionnaire. The participants' diverse roles in patient care were critical for evaluating the effectiveness and challenges of interprofessional collaboration within the hospital setting. A total of 220 participants were enrolled in this study.

Inclusion criteria were as follows: (1) being directly involved in patient care within the family medicine department and (2) having at least six months of continuous clinical experience at Unnan City Hospital in their current role. Exclusion criteria included the following: (1) administrative staff or personnel not engaged in direct patient care, (2) individuals employed at the hospital for less than six months, (3) healthcare professionals currently on extended leave during the study period (e.g., maternity, medical, or sabbatical), and (4) individuals who declined to provide informed consent or submitted incomplete questionnaire responses.

Measurements

We developed the explanatory questionnaire to examine the perceptions of other medical professionals regarding the implementation of team-based care in family medicine. As there was no research regarding the questionnaires investigating such perceptions, the questionnaire was developed through the discussion among the research team. The first and second researchers reviewed the previous articles on team-based care and constructed the following questionnaire (Table 1).

**Table 1:** The explanatory questionnaire to examine the perceptions of other medical professionals regarding the implementation of team-based care in family medicine

Question	Concept	Content
1	Better consultation	It has become easier to consult with the community care physician.
2	Beter coordination	It has become easier to coordinate work with the community care physician.
3	Understand physicians' thoughts	I have become able to understand the thoughts of the community care physician.
4	Understand patients from various perspectives	I have become able to understand patients from a multifaceted perspective.
5	Improvement of patient satisfaction	I believe patient satisfaction has improved.
6	Improvement in work efficiency	Work efficiency has improved.
7	Reduction in work-related stress	Work-related stress has decreased.
8	Reduction in working hours	Working hours have been reduced.
9	Decreased in Errors	Errors in work have decreased.
10	Positive impression of the team-based system	I have a positive impression of the introduction of the team-based system in the community care department.

To ensure the questionnaire's content validity, we consulted with three external experts in family medicine, healthcare quality improvement, and medical education who were not involved in the study. They independently reviewed the questionnaire to assess whether the items appropriately measured perceptions of team-based care, and their feedback was used to refine the wording and structure of the items. Additionally, we conducted a pilot test of the questionnaire with a small group of 10 healthcare professionals at Unnan City Hospital who were not part of the final study sample. Based on their feedback regarding clarity, relevance, and consistency, minor modifications were made to improve comprehension. Although formal statistical tests for reliability (e.g., Cronbach’s alpha) were not performed due to the small pilot sample size, this preliminary step helped establish face and content validity and improved the questionnaire's overall usability.

In this questionnaire, participants were asked to rate their level of agreement with the following statements using a five-point Likert scale from one (do not agree) to five (strongly agree). Each Likert scale question was followed by an open-ended response section, allowing participants to elaborate on their answers.

Analysis

For the quantitative analysis, descriptive statistics were first conducted to summarize the demographic characteristics of the participants, including age, gender, years of clinical experience, and professional role. Means, standard deviations (SD), and proportions were calculated for continuous and categorical variables. We employed the following statistical tests to evaluate differences in perceptions of team-based care among healthcare professionals. The Shapiro-Wilk test was used to assess the normality of continuous variables. If data were normally distributed, parametric tests were applied; otherwise, non-parametric tests were used.

Regarding the comparisons between professional groups, the chi-square test was used to determine differences in categorical responses among professional roles. Given the ordinal nature of the Likert scale responses, Kruskal-Wallis tests were conducted to compare differences across multiple professional groups. For interpretation, the five-point Likert scale responses were categorized as follows: 3> Negative perception, 3 = Neutral, 3< Positive perception. These thresholds were applied to each item to distinguish the general direction of perception among participants. While Likert scale data is ordinal and analyzed accordingly with non-parametric methods, this categorization allowed for more straightforward interpretation of trends in perception across professional groups.

We did not compute a total composite score across all questions; however, future studies may consider developing and validating a scoring system to represent overall perception. Given the ordinal nature of the Likert-scale responses, the Kruskal-Wallis test was selected as the primary statistical method to compare perceptions across professional groups. While means and standard deviations are reported for interpretability, we acknowledge the limitations of treating ordinal data as interval-scale in this context. ANOVA was not used due to the non-parametric nature of the data and the potential violation of normality assumptions. All data analyses were performed using Easy R (version 1.23; R Foundation for Statistical Computing, Vienna, Austria). Statistical significance was defined at p < 0.05.

The qualitative data of the questionnaire were analyzed through thematic analysis following the six-step approach proposed by Braun et al. [15]. The first and second researchers (RO and JO) read all responses multiple times to ensure a deep understanding of the content. Both researchers conducted open coding independently to identify meaningful segments in the data. Codes were developed based on recurring patterns in participants' responses. The initial codes were reviewed and categorized into broader themes through discussions between the researchers. Discrepancies in coding were resolved through consensus. Codes were grouped into potential themes representing key perceptions and concerns regarding team-based care. The researchers refined the themes by cross-checking against the original data to ensure coherence and accuracy. The final themes were defined, labeled, and structured into a conceptual framework.

As this study followed a convergent mixed-methods design, the quantitative and qualitative findings were analyzed in parallel and then integrated for interpretation. This approach allowed for a comprehensive understanding of how different healthcare professionals perceive the implementation of team-based care. Quantitative findings provided a broad overview of differences in perceptions across professional roles. Qualitative findings offered more profound insights into the reasons behind these differences, capturing contextual challenges and benefits of team-based care. The integration process involved comparing and contrasting themes from qualitative analysis with statistical trends from the quantitative results, ensuring a holistic interpretation of the study’s findings.

Ethical consideration

The hospital was assured that patient anonymity and confidentiality of information would not be compromised. Information related to this study was posted on the hospital website without disclosing any details about the patients. In addition, contact information for hospital personnel was posted on the website to respond to questions regarding this study. All participants were informed of the purpose of this study, and informed consent was obtained. The clinical ethics committee of the Unnan City Hospital approved this research (the approval code: 20230027).

## Results

Characteristics of the participants

A total of 220 healthcare professionals participated in this study. The participants represented a diverse cross-section of roles involved in patient care within the family medicine department at Unnan City Hospital. Most respondents were female (77.7%), and most were 41-60 years old, indicating a relatively experienced workforce. The mean duration of clinical experience was 19.75 years (SD = 11.43), while the average time working in their current role was 15.62 years (SD = 10.74). Nurses comprised the most significant professional group (70.3%), consistent with their central role in inpatient care and coordination. Therapists accounted for 14.6% of the sample, followed by medical technicians (10.5%), pharmacists (2.7%), and social workers (1.8%) (Table 2).

**Table 2:** Characteristics of the participants SD: Standard deviation

Factor	N=220
Age, year old (%)	
20-30	33 (15.0)
31-40	49 (22.3)
41-50	70 (31.8)
51-60	52 (23.6)
Over 60	16 (7.3)
Male Sex (%)	49 (22.3)
Female Sex (%)	171 (77.7)
Clinical experience, years (SD)	19.75 (11.43)
Working duration, years (SD)	15.62 (10.74)
Job (%)	
Nurse	154 (70.3)
Therapist	32 (14.6)
Technician	23 (10.5)
Pharmacist	6 (2.7)
Social worker	4 (1.8)
No answer	1 (0.4)

Differences in perception regarding the implementation of the team-based system in family medicine among medical professionals

Perceptions of team-based care varied notably across professional groups, particularly in communication, understanding of physician roles, and workload impact. Statistically significant differences were observed in multiple domains. Social workers and therapists reported the most favorable perceptions overall, especially in their ability to consult with physicians and understand their perspectives. In contrast, technicians consistently reported lower scores across several domains, including communication ease, understanding of physicians’ intentions, and reduced work-related stress. Nurses showed moderate to positive views regarding stress reduction and improved collaboration, while pharmacists expressed mixed perceptions, with some concerns related to role clarity and workflow integration.

Several key items demonstrated statistically significant intergroup differences. These included perceptions of ease of consultation with physicians (p = 0.002), understanding of physician perspectives (p = 0.032), reduction in work-related stress (p = 0.028), decreased working hours (p = 0.004), and perceived reduction in work errors (p = 0.029). Notably, social workers reported the highest positive impressions of the team-based care system (p = 0.050), reflecting their alignment with patient-centered collaborative practices. In contrast, perceptions of coordination, patient satisfaction, and work efficiency did not significantly differ across groups. The detailed distribution of mean Likert scores by professional group is presented in Table 3.

**Table 3:** Differences in perception regarding the implementation of the team-based system in family medicine among medical professionals All values are presented as mean (standard deviation) unless otherwise noted. Kruskal-Wallis tests were used to compare Likert scale responses across groups.

Questionnaire	Nurses	Therapists	Technician	Pharmacist	Social worker	P value
n	154	32	23	6	4	
Better consultation	3.38 (0.73)	3.72 (0.68)	3.00 (0.60)	3.50 (0.55)	4.00 (0.00)	0.002
Beter coordination	3.35 (0.78)	3.12 (0.34)	3.04 (0.71)	3.17 (0.41)	3.25 (0.50)	0.227
Understand physicians' thoughts	3.48 (0.90)	3.59 (0.67)	2.96 (0.88)	3.00 (0.63)	3.75 (0.50)	0.032
Understand patients from various perspectives	3.42 (0.67)	3.44 (0.62)	3.13 (0.81)	3.50 (0.55)	4.00 (0.00)	0.13
Improvement of patient satisfaction	3.14 (0.77)	3.31 (0.64)	3.22 (0.95)	3.50 (0.55)	3.25 (0.50)	0.658
Improvement in work efficiency	3.05 (0.71)	3.22 (0.71)	3.04 (0.71)	3.33 (0.52)	2.75 (0.50)	0.526
Reduction in work-related stress	3.14 (0.96)	3.19 (0.74)	2.48 (0.95)	2.83 (0.75)	3.00 (0.82)	0.028
Reduction in working hours	2.84 (0.97)	2.78 (0.75)	2.09 (0.90)	2.50 (0.84)	3.50 (0.58)	0.004
Decrease in errors	3.01 (0.74)	3.03 (0.40)	2.65 (0.78)	2.67 (1.03)	3.75 (0.96)	0.029
Positive impression of the team-based system	3.66 (0.82)	3.75 (0.62)	3.26 (1.01)	3.17 (1.17)	4.25 (0.50)	0.05

Qualitative analysis of qualitative description investigating the perceptions of medical professionals regarding the implementation of the team-based system in family medicine

Through thematic analysis of the questionnaire responses regarding the introduction of a team-based approach in the Department of Family Medicine, five main themes emerged: (1) changes in communication with physicians, (2) decision-making processes and information sharing, (3) changes in work efficiency and burden, (4) patient satisfaction and concerns, and (5) perceptions of team-based care implementation (Table 4).

**Table 4:** Five themes regarding the perceptions of medical professionals about the implementation of the team-based system in family medicine

Themes
Changes in Communication with Physicians,
Decision-Making Processes and Information Sharing
Changes in Work Efficiency and Burden
Patient Satisfaction and Concerns
Perceptions of Team-Based Care Implementation

**Figure 1:**
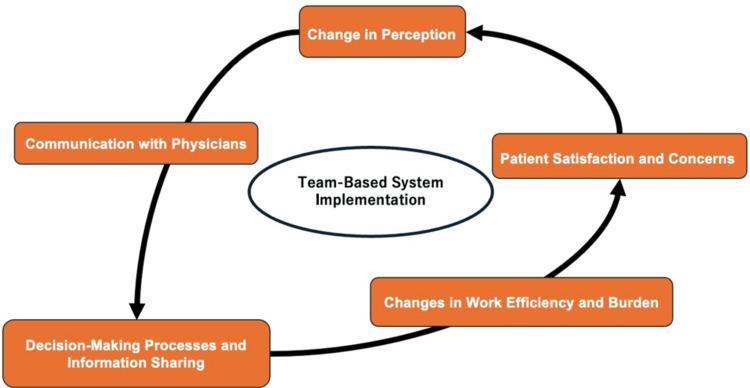
Conceptual figure of the change through the implementation of the team-based system Image Credit: Ryuichi Ohta

The team-based system improved physician accessibility and facilitated rapid decision-making in urgent cases. However, inconsistent information sharing, unclear physician roles, and an increased workload due to redundant testing were notable challenges. Additionally, patients exhibited varied reactions; some appreciated the frequent physician visits, while others experienced anxiety due to a lack of continuity in care. Addressing these challenges through structured communication protocols, standardized treatment guidelines, and improved patient education may optimize the benefits of team-based care. Figure 1 shows the conceptual figure of the thematic analysis.

Changes in Communication With Physicians

Many respondents reported that the team-based system improved their ability to communicate with physicians, citing better accessibility and responsiveness. One participant noted, "I can now consult doctors other than the primary physician, which is reassuring" (Participant 2, nurse). Another stated, "The attending physicians ask more frequently about patients, making discussions easier" (Participant 15, nurse). However, some respondents expressed concerns about inconsistencies in communication. One nurse highlighted, "Even though I can report things more easily, sometimes the primary physician is not informed by the on-call doctor, leading to confusion" (Participant 18, nurse). Another participant mentioned, "I still don’t know which doctor I should consult in certain situations" (Participant 43, pharmacist).

Decision-Making Processes and Information Sharing

Opinions varied regarding the system’s impact on decision-making. Some found it beneficial, as illustrated by one response: "Having multiple physicians involved means we get diverse perspectives, which improves treatment decisions." “Another stated, "Interdisciplinary discussions have helped form more comprehensive treatment plans" (Participant 3, nurse). Conversely, others highlighted inconsistencies in physician decision-making. One nurse remarked, "Different doctors give different orders, leading to unnecessary tests, which increases the patient’s burden" (Participant 21, nurse). Another respondent reported, "A patient with a 'Do Not Attempt Resuscitation' (DNAR) order was prescribed unnecessary tests like blood cultures, causing confusion and distress among staff" (Participant 25, nurse).

Changes in Work Efficiency and Burden

Several respondents reported increased efficiency, particularly in urgent situations. One participant noted, "I no longer have to worry about whether the primary physician is available- there is always someone I can call" (Participant 33, nurse). Another shared, "Tasks such as reporting panic values have become more streamlined" (Participant 6, nurse). However, others felt that the team-based system increased workload rather than reducing it. One nurse explained, "Multiple doctors ordering tests for the same patient leads to redundant procedures and wasted time" (Participant 70, nurse). Another noted, "Doctors give last-minute orders in the evening or at night, making it difficult to manage workload efficiently" (Participant 9, technician).

Patient Satisfaction and Concerns

Patients expressed mixed reactions to the team-based system. Some appreciated the increased medical attention, as one respondent noted: "Patients seem reassured when multiple doctors visit them every day" (Participant 90, nurse). Another observed, "Patients feel that they are being properly examined due to frequent physician rounds" (Participant 95, nurse). However, others felt that patients experienced confusion regarding their primary physician. One participant stated, "Many patients ask, ‘Who is my doctor?’ because they see different faces every day" (Participant 77, nurse). Another mentioned, "Some patients have voiced concerns that their primary doctor doesn’t visit them anymore" (Participant 11, nurse). Additionally, a respondent noted, "Some elderly patients, in particular, are not comfortable with the team-based system and prefer to have a consistent doctor they can rely on" (Participant 78, nurse).

Perceptions of Team-Based Care Implementation

While some respondents viewed the team-based approach as beneficial for physician workload distribution, others felt that the transition lacked proper structure. One participant stated, "The team-based approach is good for doctors’ work-life balance, but communication within the team is not always smooth" (Participant 25, nurse). Another remarked, "There was no clear announcement when the system was implemented, and many staff members still don’t know how it works" (Participant 89, nurse). Concerns were also raised about the lack of standardization. One respondent noted, "The lack of a unified treatment direction sometimes leads to conflicting decisions. (Participant 79, nurse). Another shared, "On-call doctors sometimes say, ‘I don’t know this patient, ask their primary doctor,’ which defeats the purpose of a team-based system" (Participant 66, nurse).

## Discussion

This study examined healthcare professionals' perceptions regarding implementing team-based care in family medicine. The findings suggest that, while team-based care improves communication, collaboration, and physician accessibility, challenges remain regarding role clarity, decision-making consistency, workload distribution, and patient continuity of care. Addressing these issues is essential for optimizing the effectiveness of team-based care in hospital settings.

One of the most significant benefits of team-based care was improved communication among healthcare professionals. Many participants, particularly nurses and therapists, reported that the system facilitated easier and more frequent consultations with physicians. This improvement in accessibility aligns with previous studies demonstrating that interprofessional collaboration enhances information exchange, leading to better-coordinated patient care [16,17]. In family medicine, where multiple disciplines are involved in patient management, efficient communication is essential for ensuring timely interventions and minimizing medical errors.

However, inconsistencies in communication were also reported. Some participants expressed difficulty determining which physician to consult, particularly in urgent situations. Others highlighted issues with fragmented information sharing between primary physicians and on-call doctors, which sometimes leads to confusion and unnecessary delays in patient management. Previous research has emphasized the importance of structured communication protocols in interprofessional settings to prevent these inefficiencies [18]. To address these challenges, hospitals should establish clear role definitions and implement standardized reporting mechanisms to ensure that all team members are consistently informed.

Interdisciplinary collaboration was perceived to improve decision-making by incorporating diverse professional perspectives. Many participants noted that having multiple professionals involved in patient care led to more comprehensive treatment plans. This finding supports prior research indicating that team-based models contribute to more holistic and well-informed clinical decisions, ultimately improving patient outcomes [19].

However, inconsistent decision-making was a significant concern. Some professionals reported that different physicians provided conflicting orders, leading to redundant or unnecessary tests. Such inefficiencies increase healthcare costs and may contribute to patient discomfort and extended hospital stays. These findings highlight the need for standardized clinical pathways to ensure consistency in decision-making while maintaining flexibility for individualized patient needs [20,21]. Developing structured guidelines and promoting interdisciplinary case discussions may mitigate these discrepancies and improve the efficiency of team-based care.

The impact of team-based care on workload was mixed among participants. Nurses and therapists felt the system improved efficiency by ensuring physicians were more readily available. This aligns with previous findings suggesting that interprofessional collaboration can streamline workflow and reduce the burden on individual providers [22,23]. Additionally, having multiple professionals involved in care was seen as beneficial in handling urgent cases more effectively.

Conversely, technicians and pharmacists expressed concerns that the system increased their workload, mainly due to redundant test orders and unclear delegation of responsibilities. Several respondents noted that multiple physicians ordering tests for the same patient created inefficiencies. Without transparent task allocation, team-based care can paradoxically increase administrative burden rather than reduce it [24]. Hospitals should address these concerns by defining task distribution clearly and implementing centralized documentation systems to prevent redundant procedures.

Patient reactions to the team-based system were varied. Some appreciated the increased attention from physicians, which reassured them about the quality of their care. Others, particularly elderly patients, expressed confusion and discomfort with seeing different doctors daily. These findings are consistent with previous studies showing that continuity of care is a key factor in patient satisfaction, especially among older adults and those with chronic illnesses [25].

To address these concerns, it may be beneficial to designate a primary physician within each team to maintain continuity while allowing for interdisciplinary input. Additionally, patient education initiatives should be implemented to clarify the roles of different team members, reducing confusion about who is responsible for their care [26,27]. Clear communication with patients regarding how the team-based system operates can enhance their understanding and acceptance of the model.

The findings revealed that perceptions of team-based care varied by profession. Social workers and therapists had the most positive views, likely due to their prior experience working in interdisciplinary settings. In contrast, technicians and pharmacists were more critical, possibly because of workflow disruptions or increased workload. These discrepancies highlight the need for tailored interventions to address the specific concerns of each professional group [28].

A key concern raised was the lack of structured implementation. Many participants reported that the transition to team-based care was poorly communicated, leading to uncertainty among staff. Successful implementation of such systems requires a structured approach, including clear guidelines, role definitions, and adequate training for all healthcare professionals [29]. Orientation programs and ongoing support can facilitate smoother integration and higher acceptance of team-based models.

This study has several limitations. First, it was conducted in a single hospital, which may limit the generalizability of the findings to other settings. Future research should include multiple institutions to provide a broader perspective on team-based care implementation. Second, the study relied on self-reported data, which may be subject to response bias. Participants may have provided socially desirable responses rather than entirely objective assessments. Triangulating survey responses with observational data or patient outcome measures could enhance the robustness of findings. Finally, the study did not assess long-term outcomes of team-based care, such as changes in patient health status or hospital efficiency over time. Longitudinal studies are needed to evaluate the sustained impact of interprofessional collaboration in family medicine.

## Conclusions

This study found that healthcare professionals received team-based care in family medicine positively, though perceptions varied across roles. Improved communication, interdisciplinary collaboration, and physician accessibility were identified as key benefits. However, challenges such as inconsistent information sharing, increased workload for some professionals, and patient concerns about continuity of care were also highlighted. More explicit communication strategies, standardized workflows, and patient-centered approaches are necessary to optimize the implementation of team-based care. Future research should explore long-term patient outcomes and the impact of structured implementation strategies on healthcare efficiency.
